# A new *Globba* with large white floral bracts from Peninsular Malaysia

**DOI:** 10.3897/phytokeys.73.9737

**Published:** 2016-10-25

**Authors:** Yen Yen Sam, Halijah Ibrahim

**Affiliations:** 1 Forest Research Institute Malaysia, 52109 Kepong, Selangor, Malaysia; 2 Institute of Biological Sciences, Faculty of Science, University of Malaya, 50603 Kuala Lumpur, Malaysia

**Keywords:** Endemic, ginger, Globbeae, taxonomy, Terengganu, Zingiberaceae

## Abstract

*Globba magnibracteata* Y.Y.Sam, **sp. nov.** is described and illustrated. Colour plates, a preliminary conservation assessment and a discussion of its closely related taxa are provided.

## Introduction

*Globba* is one of the largest genera in the Zingiberaceae family with over 100 species mostly found in the Indo-Chinese monsoon region. In Peninsular Malaysia, fourteen species, five subspecies, eight varieties and a natural hybrid have been documented thus far (Newman et al. 2004; Sam et al. 2012). The genus is a common ground herb in the peninsula’s tropical evergreen rainforest. Ridley (1924) provided the first taxonomic account of the Peninsular Malaysian species. He has identified twenty one species of which twelve were named by him. Holttum (1950), in his revision on the Zingiberaceae of Peninsular Malaysia, has changed some of Ridley’s species to varietal rank or synonymised them and greatly reducing the number to ten species and ten varieties. A detailed cytological and morphological study by Lim (1972) has discovered a number of new specific and infraspecific taxa, bringing the total to twelve species, five subspecies, eight varieties and one natural hybrid. Several new taxa continued to be named in the following years as plant collecting ventured further into the remote forested areas (Weber 1991; Ibrahim and Larsen 1995; Sam et al. 2012). Here in Peninsular Malaysia, we discovered another new species from the interior of Terengganu. This plant has remarkable large, white floral bracts which are well spaced on the long arching inflorescence. These strongly reflexed sterile bracts are the largest amongst the peninsular species and this feature clearly distinguished it from others.

The current classification of the genus recognises three subgenera, seven sections and two subsections based on the structure of the anther appendage (Williams et al. 2004). In Peninsular Malaysia, only the subgenus Globba, with four appendages and the two-appendage *Ceratanthera* have been recorded, none being from subgenus Mantisia. Twelve species, including the new *Globba magnibracteata* belong to the subgenus Globba section Sempervirens, while only three are placed in the subgenus Ceratanthera.

## Taxonomy

### Globba magnibracteata

Taxon classificationPlantaeZingiberalesZingiberaceae

Y.Y.Sam sp. nov.

urn:lsid:ipni.org:names:77158314-1

Figures 1 , 2

#### Diagnosis.

*Globba magnibracteata* is similar to *Globba albobracteata* N.E.Br. where both are placed in the subgenus Globba section Sempervirens. They have the same vegetative morphologies and inflorescence structure but differ in having wide spreading or strongly deflexed white sterile bracts versus the green appressed sterile bracts of *Globba albobracteata*. The elliptic fertile bracts of *Globba magnibracteata* are smaller (1.1–1.2 cm long) compared to the obovate bracts of *Globba albobracteata* which are about 3 cm long. The cincinnus stalk of *Globba magnibracteata* is also shorter (less than 1 cm) than that of *Globba albobracteata* (2–4 cm). *Globba magnibracteata* has bulbils with many roots and one bamboo-like shoot distinct from the one-root-one-shoot bulbils in *Globba albobracteata*.

#### Type.

MALAYSIA. Peninsular Malaysia, Terengganu, Jengai Forest Reserve, Compartment 5, 4°39.59'N, 103°05.05'E, 21 April 2009, Sam & Aidil FRI 68959 (holotype: KEP; isotypes: E, KLU, SAN, SING)

#### Description.

Rhizomatous herb, evergreen, 30–70 cm tall, in small clumps of 3–4 leafy stems. Rhizome c. 5 mm diameter, not tuberous. Leafy stems bend on a large curve, basal stem slightly swollen; base to first leaf (18–) 25–32 cm long; first leaf to the uppermost leaf sheath (32–) 42–54 cm long; bladeless sheaths 3–4, pubescent, persistent, lower sheaths purplish when young; leaf sheath pubescent; ligule truncate, pubescent, persistent, 1–2 mm long; leaves 9–13, 3–6 cm apart, almost sessile; lamina narrowly ovate to elliptic, (14)17–22 × (3.8) 4.3–6.7 cm, adaxial dark green with slightly raised lateral veins, glabrous, abaxial pale green, pubescent, base cuneate, apex attenuate with long acumen. Inflorescence terminal, 13.2–17 cm long, bent downwards in a very broad curve, rachis pointing perpendicularly down; peduncle 9.5–11 cm long, green, pubescent, with 4–6 large sterile bracts positioned at the middle part only; sterile bracts elliptic-oblong, largest 4.5–6.5 × 0.7–1.2 cm, white, pubescent, persistent, spreading to strongly reflexed, very lax; rachis 2.7–7 cm long, axis green, pubescent, 7–17 cincinni, lax; fertile bracts elliptic, 11–12 × c. 4 mm, white, pubescent, spreading to reflexed, persistent; cincinni 6–22 mm long, pubescent, up to 8 mm long to first flower; bracteoles boat shaped, 3–4 mm long, orange. Flowers orange, up to 14 flowers on each cincinnus; pedicel c. 1 mm, green, pubescent; calyx tubular, 3–4 mm long, pubescent, apex trilobed; corolla tube 14–15 mm long, pubescent; dorsal corolla lobe c. 5 mm long, concave, apex hooded, abaxial hairy; lateral corolla lobes c. 4 × 2.5 mm, broadly elliptic, apex rounded, margin curved in when fresh, abaxial hairy; lateral staminodes narrowly ovate, c. 5 × 1.5 mm, same length as corolla lobes, spreading, apex acuminate; labellum 5–6 mm long, 2 brown spots in the centre, base bifid, c. 4 mm wide, lobes divergent. Stamen filament c. 17 mm long; anther c. 2 mm long, with 4 appendages; appendages triangular, c. 2.5 × 1 mm, spreading wide. Ovary c. 2 mm long, orange, pubescent, unilocular; stigma c. 1 × 1 mm, clavate, ciliate, ostiole transverse, facing upwards; epigynous glands linear, 2, c. 4 mm long. Fruits globose, glabrous, pale green when young; seeds not observed. Bulbils at the position of last flower in cincinni, many roots and shoots but only one developed into bamboo-like shoot.

**Figure 1.**
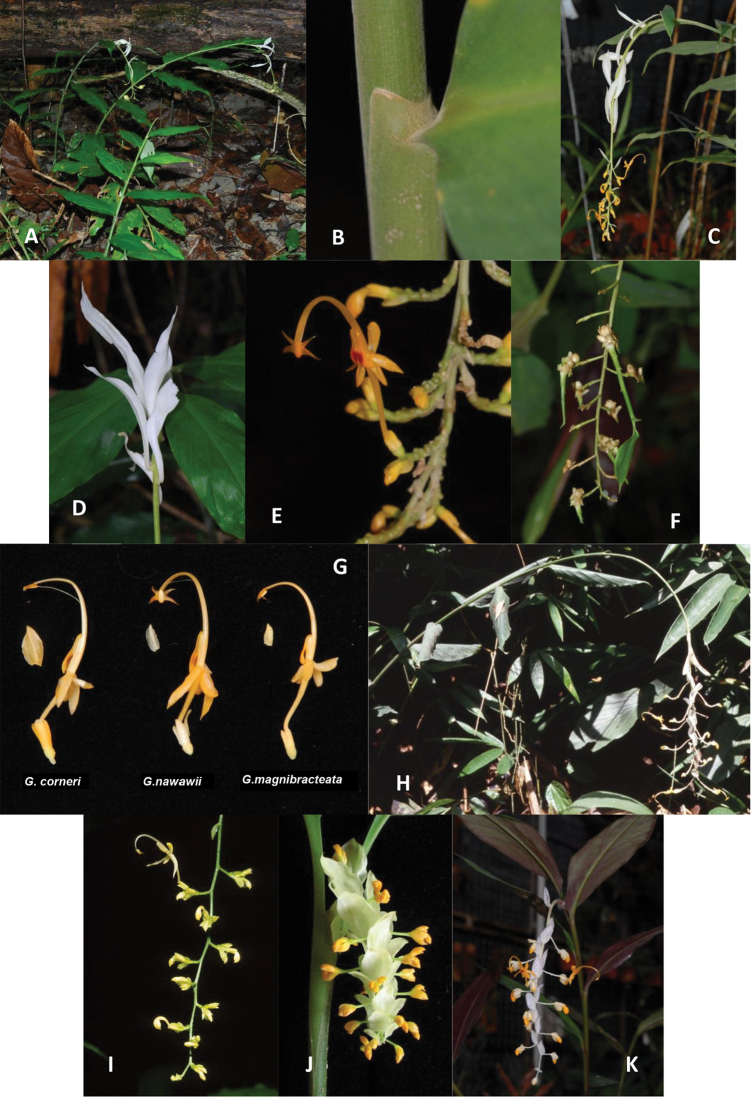
**A–D** *Globba magnibracteata* **A** Habit **B** Ligule **C** Inflorescence **D** Sterile floral bracts **E** Flower **F** Bulbils **G** Flowers of *Globba corneri*, *Globba nawawii* and *Globba magnibracteata* **H** *Globba albobracteata* **I** *Globba cernua* **J** *Globba corneri* **K** *Globba nawawii*. (Photographs **A–G, I** and **K** by Y.Y. Sam, **H** by A. Takano, **J** by Y.M. Chan).

**Figure 2.**
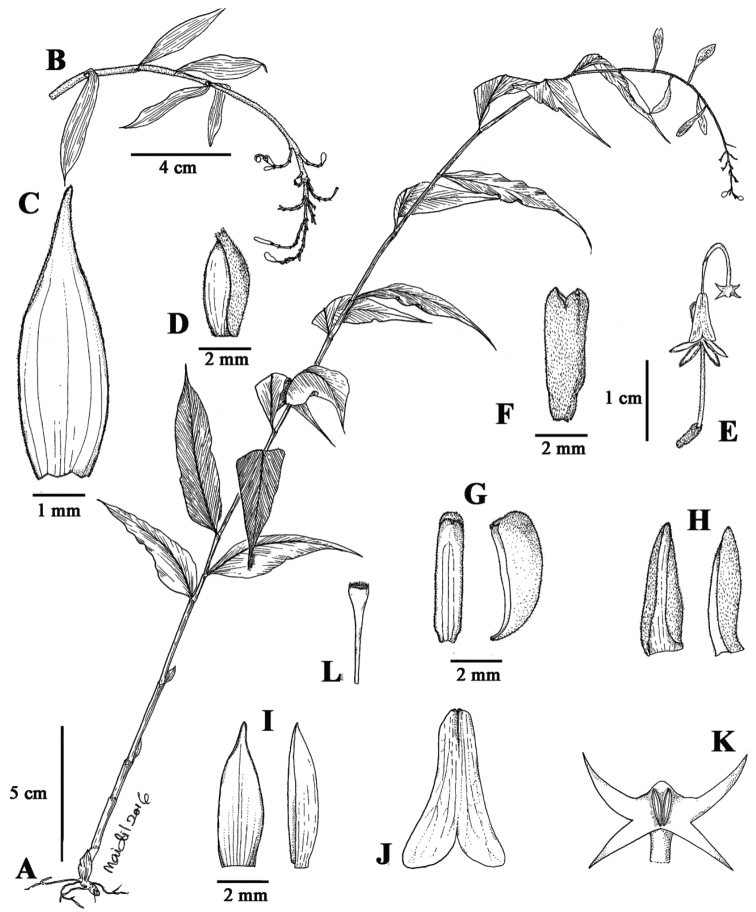
*Globba magnibracteata* Y.Y.Sam **A** Habit **B** Inflorescence **C** Sterile bract **D** Bracteole **E** Flower **F** Ovary and calyx **G** Dorsal corolla lobe in front and side view **H** Lateral corolla lobe in ventral and side view **I** Lateral staminodes in ventral and side view **J** Labellum **K** Anther appendages **L** Stigma. Drawn by M.N. Aidil from Sam & Aidil FRI 68959 (KEP).

#### Etymology.

The epithet is derived from Latin and refers to the large (*magnus*) floral bract (*bractea*).

#### Distribution and ecology.

*Globba magnibracteata* is only known from Jengai Forest Reserve, Peninsular Malaysia. The plants were found scattered on the shady and moist forest floor with a thick humus layer in the lowland dipterocarp forest, a tropical evergreen rainforest.

#### Preliminary conservation assessment.

Critically Endangered, CR B2ab(iii). *Globba magnibracteata* is only found in Compartment 5 in the Jengai Forest Reserve which is a production forest subjected to selective logging on a rotation basis. Timber harvesting will inevitably and adversely affect the quality of the forest, especially the niche environment where the plants are to be found. The population is also very small, less than 20 mature individuals being encountered at the site. Although several extensive botanical collections in other compartments in the same reserved forest were undertaken, no *Globba magnibracteata* was sighted. The area of occupancy for *Globba magnibracteata* is only 4 km^2^ plus its small population, thereby qualifying the species to be listed in the Critically Endangered category (IUCN 2016).

#### Notes.

*Globba magnibracteata* closely resembles *Globba albobracteata* from Sumatra, Indonesia. Both have about 5–6 pairs of leaves spaced widely on the slender leafy stems and are also similar in lamina shape and size, inflorescence structure and flower colour. The differences lie in the sterile and fertile bracts, cincinni and floral parts. *Globba magnibracteata* has large white sterile bracts, which are wide spreading or deflexed, visible even at a far distance. The green sterile bracts of *Globba albobracteata* are of similar size but they are appressed and overlapped on the peduncle making them not readily noticeable. The fertile bract is another distinguishable feature, *Globba magnibracteata* having elliptic and smaller (1.1–1.2 cm) bracts compared to the obovate bracts of *Globba albobracteata*, which are about 3 cm long. For the cincinni, the stalk of *Globba magnibracteata* is clearly shorter, measuring less than 1 cm whereas it is 2–4 cm in *Globba albobracteata*. In addition, the corolla lobes, lateral staminodes and labellum of *Globba magnibracteata* are consistently smaller compared to *Globba albobracteata* (Table 1).

**Table 1.** Comparison of the morphological characters of *Globba magnibracteata*, *Globba albobracteata*, *Globba cernua*, *Globba corneri* and *Globba nawawii*.

Characters	*Globba magnibracteata*	*Globba albobracteata* (Takano & Okada, 2003)	*Globba cernua* (Holttum, 1950; Takano & Okada, 2003)	*Globba corneri* (Weber, 1991)	*Globba nawawii* (Ibrahim & Larsen, 1995)
Leaf number & position	9–13; spaced along leafy stem	9–11; spaced along leafy stem	4–7; spaced along leafy stem	2; terminal	3–5; crowded in upper stem
Petiole (cm)	almost sessile	sessile	to c. 4 mm	6–7	sessile
Lamina size (cm)	(14)17–22 × (3.8) 4.3–6.7	13–19 × 3–6	15–19 × 3.5–5	30 × 10	c. 14.5 × 4.5
Inflorescence length (cm)	13.2–17	—	8–20	c. 8	13–21
Peduncle length (cm) & colour	9.5–11; light green	20–35; pale green	c. 12.5; green	0–3; pale green	6–10; whitish to light purplish red
Rachis (cm)	2.7–7	up to 15	5–10	3–5	7–11
Size (cm) and shape of sterile bracts	4.5–6.5 × 0.7–1.2; elliptic-oblong	5–6 cm long; lanceolate-ensiform	0.7 × 0.25; ovate to elliptic	2(–2.5) × 1(–1.5); ovate or broadly lanceolate	1.2–1.4 × 0.5–0.7; ovate-acuminate
Colour & structure of sterile bracts	white; spreading to strongly reflexed	green; imbricate	green; not imbricate	white; strongly reflexed	white; reflexed
Size (mm) and shape of fertile bracts	11–12 × c. 4; elliptic	30 × 10; obovate	8 mm long; ovate	20(–25) × 10(–15); ovate or broadly lanceolate	12–14 × 5–7; ovate-acuminate
Colour & structure of fertile bracts	white; spreading to reflexed	white; spreading to reflexed	yellow green; deflexed	white; strongly reflexed	white; spreading to strongly reflexed
Colour of flowers	orange	orange	pale yellow	orange	orange
Length of staminodes vs. corolla lobes	same	slightly longer	twice	twice	twice

*Globba* c*ernua* Baker is another species closely related to *Globba magnibracteata*. Both grow in small clusters with 2–4 leafy shoots and their long inflorescences hang downwards in a very broad curve. Nevertheless, the distinctly white, large sterile bracts and the orange flowers of *Globba magnibracteata* immediately separate it from the small green bracts and pale yellow flowers of *Globba cernua*. Both sterile and fertile bracts of *Globba magnibracteata* remain attached to the inflorescence but in *Globba cernua*, the bracts are shed at the early stage of flowering. Other differences were also observed upon closer examination, such as the number of leaves and size of the staminodes (Table 1).

Among the *Globba* species in Peninsular Malaysia, there are two species with conspicuous white bracts: *Globba corneri* A.Weber and *Globba nawawii* H.Ibrahim & K.Larsen, which look similar to *Globba magnibracteata* but there are several features which distinguish them (Table 1). *Globba corneri* differs from *Globba magnibracteata* in the following characteristics: leafy stems with two leaves crowded at terminal, short peduncle that bend abruptly downwards, large white fertile bracts and the lateral staminodes are twice the length of the corolla lobes. For *Globba nawawii*, it has fewer leaves, longer rachis, smaller sterile and fertile bracts and lateral staminodes twice the length of the corolla lobes compared with those *Globba magnibracteata*.

There are several Thai globbas with large showy floral bracts such as *Globba candida*, *Globba laeta*, *Globba siamensis*, *Globba winittii*, but they are not allied to *Globba magnibracteata*. These plants thrive in the seasonal forest and their leafy parts die back during the dry season leaving the rhizome dormant underground. It is not possible to find these species in the evergreen forest of Peninsular Malaysia where *Globba magnibracteata* grows.

## Supplementary Material

XML Treatment for Globba magnibracteata
